# National Divergences in Perinatal Palliative Care Guidelines and Training in Tertiary NICUs

**DOI:** 10.3389/fped.2021.673545

**Published:** 2021-07-14

**Authors:** Antonio Boan Pion, Julia Baenziger, Jean-Claude Fauchère, Deborah Gubler, Manya J. Hendriks

**Affiliations:** ^1^Department of Neonatology, University Hospital Zurich, University of Zurich, Zurich, Switzerland; ^2^Department of Health Sciences and Medicine, University of Lucerne, Lucerne, Switzerland; ^3^Pediatric Palliative Care, University Children's Hospital Zurich, University of Zurich, Zurich, Switzerland; ^4^Clinical Ethics, University Hospital Zurich, University of Zurich, Zurich, Switzerland

**Keywords:** perinatal palliative care, neonatal, life-limiting diagnosis, neonatal healthcare professionals, guidelines, continued training, education

## Abstract

**Objectives:** Despite established principles of perinatal palliative care (PnPC), implementation into practice has shown inconsistencies. The aim of this study was to assess PnPC services, examine healthcare professionals (HCPs) awareness and availability of PnPC guidelines, and describe HCPs satisfaction with PC and guidelines.

**Material and Methods:** A nationwide survey was conducted in Swiss tertiary NICUs between April-November 2019. Data were examined by descriptive statistics and linear regression models.

**Results:** Overall response rate was 54% (65% physicians; 49% nurses; 72% psychosocial staff). Half of professionals (50%) received education in PC during their medical/nursing school, whereas 36% indicated they obtained further training in PnPC at their center. PnPC guidelines were available in 4/9 centers, with 68% HCPs being aware of the guideline. Professionals who had access to a PnPC team (*P* = 0.001) or were part of the nursing (*P* = 0.003) or psychosocial staff (*P* = 0.001) were more likely aware of having a guideline. Twenty-eight percent indicated being satisfied with PC in their center. Professionals with guideline awareness (*P* = 0.025), further training (*P* = 0.001), and access to a PnPC team (*P* < 0.001) were more likely to be satisfied, whereas HCPs with a nursing background (*P* < 0.001) were more likely to be dissatisfied. A majority expressed the need for a PnPC guideline (80%) and further PC training (94%).

**Conclusion:** This study reveals lacking PnPC guidelines and divergences regarding onsite opportunities for continued training across Swiss level III NICUs. Extending PnPC guidelines and training services to all centers can help bridge the barriers created by fragmented practice.

## Introduction

Despite major advances in perinatal medicine, nearly 40% of all childhood deaths occur in the first 4 weeks of life (1, 2). Future improvements in prenatal diagnostics will lead to an increased detection of potentially life-limiting conditions. Some life-limiting conditions occur intra-partum or are diagnosed postnatally (3, 4). For all of the described conditions, the role of palliative care is becoming more significant (5, 6). The verdict that one's child may be critically ill, extremely premature, or born with a life-threatening congenital anomaly represents challenging news for parents (7).

In such burdensome situations, parents and neonatal healthcare professionals (HCPs) are faced with a series of questions on how to proceed regarding the provision of life-sustaining measures. Prognostic uncertainty can add further complexity to decision-making (8). It is often uncertain whether a baby will survive or die *in utero*, during labor, or postnatally after minutes, hours, days or even weeks. Parents who face such decisions can experience high psychological, spiritual and social distress (9). These critical decisions may impact on everyone involved, hence, HCPs who are competent in perinatal palliative care (PnPC) play a vital role in mitigating adverse consequences for families.

PnPC involves a comprehensive supportive care for the affected child and their families by a multidisciplinary team ensuring highest comfort and quality of life for all involved (10, 11). This care includes end-of-life (EOL) care for the child and extends into the bereavement period. While there is a growing knowledge about key elements of PnPC, and although recommendations are emerging (12, 13), its implementation is still hampered by inconsistencies (14–16). With a growing population of critically ill fetuses and neonates with highly complex needs (17), it is essential to describe the current state of the field and the opportunities that allow for the development and implementation of high-quality PC in perinatal medicine (18).

Furthermore, as PnPC services are expanding nationwide, the level of knowledge and satisfaction that neonatal HCPs have in providing PnPC is important. To date, only limited data exists on the needs of neonatal HCPs regarding PnPC (19–23). Moreover, little is known about the PnPC services offered to critically ill patients and their families in Switzerland (9, 24). Experts in Switzerland have called for the need to analyze existing services and resources in order to derive new measures to promote PC for this vulnerable patient population (25).

Therefore, the three aims of this study were (1) to describe PnPC services, education and training; (2) to illustrate availability and awareness of PnPC guidelines, and (3) to assess satisfaction with PC in general and in particular with PnPC guidelines at the participating sites.

## Methods

### Design

In this study, we conducted a cross-sectional electronic survey among HCPs involved in PnPC in all nine-level III neonatal intensive care units (NICUs) in Switzerland. We followed the Checklist for Reporting Results of Internet Surveys (CHERRIES) (26).

### Participants and Procedure

All HCPs (neonatologists, neonatal nurses and psychosocial staff members – including psychiatrists, psychologists, and social workers) who worked in one of the nine level III NICUs in Switzerland were eligible and invited to participate. First, members from the Swiss Neonatal End-of-Life (SN-EOL) Study Group were informed about the study and indicated their willingness to serve as local study investigators. Second, contact information for all eligible HCPs was provided by local study investigators. Eligible HCPs received study information and a personal e-mail invitation (including purpose and timeframe) to participate in the closed survey using SosciSurvey (27). Two reminders were sent (2 and 4 weeks). Data collection took place between April-November 2019. SosciSurvey allows for efficient download importing to both Atlas.ti (28) and Stata 16.1. (StataCorp LP, College Station, TX).

### Measurements

After a review of the literature, the survey was developed, in which some questions were adopted from Haug et al. (16). The survey included socio-demographic questions (e.g., gender, profession) and questions about perinatal services, availability and awareness of PnPC guidelines and satisfaction. The survey was designed including a logic branch (i.e., adaptive questioning), in which specific questions are only conditionally displayed based on responses to other items. To identify the units' PnPC guideline, the local study investigators were asked to provide this information, which was converted into a code to indicate guideline availability. The German questionnaire was translated to French, back-translated, and pilot-tested by a panel of PnPC experts. An English version of the survey can be found in Supplementary Material 1. The online survey consisted of 55 questions and included 4 open-ended questions (average response time: 8.2 min, 95% CI: 7.9–8.5 min).

### Data Analyses

Descriptive analyses were performed for all aims. Additionally, we conducted Pearson's chi-squared tests to analyze associations between participant characteristics and PC education and PnPC training (aim 1). A multivariable linear regression model was carried out to identify characteristics associated with awareness of PnPC guidelines (aim 2) and HCPs satisfaction with PnPC guidelines (aim 3). For aim 2, associations were only tested among participants of hospitals with an available guideline. The multivariable linear regression model used a multilevel approach with random intercepts, constant slopes, and hospital as the group variable to account for clustering. We first tested associations in univariable regression regarding: profession (physician, nurses, psychosocial staff members), language region (German/French), PC education during medical school (yes/no), further training in PnPC at center (yes/do not know/no), access to perinatal or pediatric PC team (yes/do not know/no), work experience (little: ≤ 6 years, moderate: 7–12 years, extensive: ≥13 years), and awareness of guidelines (yes/do not know/no, for aim 2 only). Characteristics were then included as explanatory variables in the final multivariable model if associated with the respective outcome in univariable multilevel regression (threshold P < .05). Statistical analyses were carried out using Stata 16.1.

Qualitative open-ended responses regarding satisfaction with PC (aim 3) were analyzed using the principles of thematic analysis to provide insights in what can be improved with ATLAS.ti 8 (28). First, an initial coding scheme was developed guided by the rationale of our third aim (deductive) (MH). Second, preliminary codes were generated through coding of the data (ABP, MH). Third, identified codes were reviewed and the coding scheme was refined with codes that were grounded in the data (inductive). Discrepancies were resolved through repeated discussion (ABP, MH, DG). Finally, the codes were categorized into over-arching themes. To ensure accuracy of participants' quotes, back-to-back translation was performed (29). Representative quotes were selected and are presented throughout the manuscript.

### Ethics

A declaration of no objection was issued by the Ethics Commission of the Canton of Zurich (Study ID: 2019-00176). Participation was voluntary and not reimbursed, respondents were asked for consent to the study through returning the completed questionnaire, and responses were anonymized prior to analysis. The study was conducted according to the International Committee of Medical Journal Editors guidelines.

## Results

### Study Sample

Out of 804 eligible HCPs from nine Swiss tertiary NICUs, 6 participants were excluded after not answering any question except the consent page, and further 15 HCPs refused participation without providing any reason (28%), or stating either no interest (28%), no time (25%) or other reasons (19%). This led to an overall response rate of 54% with 436 HCPs participating to the online survey with 106 physicians (65%), 297 nurses (49%) and 33 psychosocial staff members (72%). A majority of HCPs had more than 12 years of professional experience (40%), and the majority of HCPs (84%) worked in the German-speaking region of Switzerland (Table 1).

**Table 1:** Characteristics of study population in the HCP-PerinatPC survey.

**Total *N* = 436**	***n* (%)**
**Language region**	
German	365 (83.7)
French	71 (16.3)
**Gender**	
Male	52 (11.9)
Female	382 (87.6)
Other	2 (0.5)
**Age at study**	
<30 years	75 (17.2)
30–39 years	151 (34.6)
40–49 years	119 (27.3)
>50 years	91 (20.9)
**Own children**	
Yes	216 (49.5)
No	218 (50.0)
Not filled in (i.e., missing)	2 (0.05)
**Religion**	
Catholic	153 (35.1)
None	148 (33.9)
Protestant	114 (26.2)
Muslim	6 (1.4)
Other	15 (3.4)
**Profession**	
Physician	106 (24.3)
Nurse	297 (68.1)
Psychosocial staff	33 (7.6)
**Leading position (*****n*** **=** **403)**^a^	
Yes	103 (25.5)
No	250 (62.0)
Not filled in (i.e., missing)	50 (12.4)
**Country of degree**	
Switzerland	326 (74.7)
Abroad	110 (25.3)
**Working experience**	
Little work experience ( ≤ 6 years)	150 (34.4)
Moderate work experience (7–12 years)	112 (25.7)
Extensive work experience (≥13 years)	174 (39.9)

a *Physicians and nurses only*. *N.B. percentages might not add up to 100% due to rounding*.

### Aim 1. Perinatal Palliative Care Services, Education, and Training

In the 12 months prior to the survey, 81% of HCPs had treated neonates with a palliative diagnosis, with an average of 1–5 cases (53%) per year (Table 2). Half of HCPs (56%) self-reported having access to a pediatric PC team in their center, whereas 42% reported having access to a PnPC team. Other PnPC services available to families were follow-up consultations (83%), grief counseling (75%), and PnPC brochures (30%). For the staff, moral distress counseling was mostly available (62%). Half of HCPs (50%) received PC education in their curriculum. Regarding further training in PnPC at their center, more than one third of HCPs (36%) indicated that they had been offered a training, whereas 33% were not offered training, and 28% did not know. Further training mainly entailed courses (67%), workshops (13%), and lectures (12%). A large majority of HCPs (94%) expressed the need for further training in PnPC in their center.

**Table 2:** Perinatal palliative care services in Swiss neonatal intensive care units.

	**Total *N* = 436 (100%)**
	***n* (%)**
**Number of PnPC patients in the last 12 months**	
None	72 (16.5)
1–5	229 (52.5)
5–10	94 (21.6)
10–15	21 (4.8)
>15	9 (2.1)
Not answered	11 (2.5)
**PnPC Team**	
Yes	182 (41.7)
No	165 (37.8)
Do not know	64 (14.7)
Not answered	25 (5.7)
**Pediatric PC Team**	
Yes	246 (56.4)
No	96 (22.0)
Do not know	69 (15.8)
Not answered	25 (5.7)
**Grief counseling**	
Yes	325 (74.5)
No	33 (7.6)
Do not know	50 (11.5)
Not answered	28 (6.4)
**Responsible for grief counseling (*****n*** **=** **325)**^a^	
Spiritual caregiver	173 (21.8)
Reference nurse	149 (18.5)
Neonatologist	137 (17.2)
Psychologist	109 (13.7)
Bereavement nurse	77 (9.7)
Psychiatrist	36 (4.5)
Midwife	26 (3.2)
Pediatrician	24 (3.0)
Others	32 (4.0)
Do not know	37 (4.5)
**PnPC brochure/literature for families**	
Yes	129 (29.6)
No	138 (31.7)
Do not know	139 (31.9)
Not answered	30 (6.9)
**Follow-up consultation**	
Yes	362 (83.0)
No	4 (0.9)
Do not know	41 (9.4)
Not answered	29 (6.7)
**Responsible for follow-up (*****n*** **=** **362)**^**a**^	
Neonatologist	295 (37.4)
Reference nurse	220 (27.8)
Spiritual caregiver	58 (7.4)
Pediatrician	55 (7.0)
Psychologist	51 (6.5)
Psychiatrist	20 (2.5)
Midwife	17 (2.2)
Others	44 (5.6)
Do not know	29 (3.7)
**Moral (dis)tress counseling for care team**	
Yes	268 (61.5)
No	71 (16.3)
Do not know	67 (15.4)
Not answered	30 (6.9)
**PC education in curriculum**	
Yes	217 (49.7)
No	209 (47.9)
Do not know	10 (2.3)
**PnPC further training at center**	
Yes	158 (36.2)
No	142 (32.6)
Do not know	124 (28.4)
Not filled in (i.e., missing)	12 (0.03)

*PC, Palliative Care; PnPC, Perinatal Palliative Care*.

a *Multiple answers possible*.

*N.B. percentages might not add up to 100% due to rounding*.

We found several associations between HCPs' characteristics and PC education or PnPC training (Supplementary Table 1). First, regarding PC education, we found that HCPs who were younger at time of study (*P* < 0.001), had little or moderate working experience (*P* < 0.001), or did not have children (*P* < 0.001) were more likely to have received PC education in their curriculum. Furthermore, HCPs with a nursing background (*P* < 0.001) more often received PC education, whereas HCPs in a leading position (*P* < 0.001) had not. Second, regarding availability of further PnPC training, we found that training was more commonly offered in German than in French-speaking areas of Switzerland (*P* < 0.001). In addition, HCPs with older age at time of study (*P* < 0.001) and with more working experience (*P* < 0.002) more often reported to have received PnPC training at their center.

### Aim 2. HCPs Availability and Awareness of Perinatal Palliative Care Guidelines

PnPC guidelines were available in 4 out of 9 centers. Among HCPs from hospitals with a guideline (*n* = 236), a majority of HCPs (68%) were *accurately* aware of having one. However, one-third of HCPs were not; they either did not know (17%), or they thought there was no guideline in their center (14%). Correspondingly, among HCPs from hospitals without a guideline (*n* = 200), 39% of HCPs were *accurately* aware of *not* having a guideline. But two-thirds of HCPs were not aware. Across these 5 centers, one-third of HCPs did not know whether their center had a guideline (29%), whereas another third reported having a guideline even though their center had none (30%).

According to HCPs with a PnPC guideline topics that were often included were psychological and social support (84%), pastoral care (82%), comfort care (79%), and withholding resuscitation (73%, Supplementary Figure 1).

In the multilevel multivariable regression analysis (Table 3), we found some characteristics associated with awareness of available PnPC guidelines among HCPs. First, HCPs with access to a PnPC team (*b* = 0.33, *P* = 0.001) were more likely aware of having an available PnPC guideline in their center. Second, HCPs with a nursing background (*b* = 0.29, *P* = 0.003) or psychosocial staff (*b* = 0.48, *P* = 0.001) were more likely aware of having an available PnPC guideline.

**Table 3:** Characteristics associated with awareness of perinatal palliative care guidelines.

**Total *N* = 223^a^**	**b**	***P***	**95% CI**
**Received PnPC further training at hospital**
No (reference)			
Do not know	0.04	0.738	(−0.18–0.25)
Yes	0.07	0.469	(−0.13–0.27)
**Access to PnPC team**
No (reference)			
Do not know	0.26	0.034	(0.02–0.50)
Yes	0.33	**0.001**	(0.15–0.52)
**Profession**
Physician (reference)			
Nurse	0.29	0.003	(0.10–0.48)
Psychosocial staff	0.48	**0.001**	(0.19–0.78)

*PnPC, Perinatal palliative care; P, p-value; 95%CI, 95% confidence interval; b: Unstandardized beta coefficient. The bold values indicate statistically significant p-values*.

a *13 answers excluded from multilevel multivariable analysis due to missings in variables*.

### Aim 3. Satisfaction With Palliative Care and Perinatal Palliative Care Guidelines

#### Provision of Palliative Care to Patients and Their Families

One third of HCPs (28%) were satisfied with the PC that was provided to patients and their families in their center, while 49% were partially satisfied, and 12% were dissatisfied.

Analysis of the open-ended questions revealed a nuanced understanding of (dis)satisfaction. One nurse with ≥13 years of working experience who was satisfied with PC considered that it was: “*Very well embedded in clinical practice, but still a lot of resistance to the topic, especially among doctors*” (No. 528). The presence or lack of certain resources and/or structures were often the main reason for HCPs' (dis)satisfaction. For example, the availability of PC concepts or guidelines, further PnPC training, specialized PC teams or experts, and (no) time pressure were the most referred barriers or facilitators for a well-established PC at the center. As one participant summarized; “*There are no resources for someone with expert skills in specialist palliative care on the ward; aftercare for the core healthcare team in stressful situations is unsatisfactory; doctors only partly deal with the latest findings in neonatal palliative care; and training for the healthcare team has limited resources*.” (Nurse, 13–20 years of work experience, No. 581). In addition, HCPs were satisfied when there was excellent team work and motivation within the team. The most common reason for HCPs to be dissatisfied with PC in their center was that colleagues equated PC to EOL care; e.g., “*Unfortunately, we always wait far too long before a patient is ‘switched' from curative to palliative care, usually only when death is imminent*.” (Nurse, ≥13 years of work experience, No. 528). A further reason given by HCPs was the lacking standard of care in PC. Some HCPs would properly support patients and parents, whereas others would not. However, (dis)satisfaction was neither black nor white and HCPs aimed to overcome the limitations present in their centers.

Our multilevel regression analysis (Table 4) showed that HCPs were more likely to be satisfied with PC when they reported having a guideline (*b* = 0.21, *P* = 0.025), having received further training in PnPC at the center (*b* = 0.26, *P* = 0.001), and had access to a PnPC team (*b* = 0.30, *P* < 0.001). However, HCPs with a nursing background (*b* = −0.31, *P* < 0.001) were more likely to be dissatisfied with PC.

**Table 4:** Characteristics associated with satisfaction with palliative care.

**Total *N* = 384^a^**	**b**	***P***	**95% CI**
**Awareness of guidelines**			
No (reference)			
Do not know	0.04	0.688688	(−0.14 to 0.21)
Yes	0.21	**0.025**	(0.03 to 0.40)
**Received PnPC further training at hospital**			
No (reference)			
Do not know	0.13	0.111	(−0.03 to 0.28)
Yes	0.26	**0.001**	(0.11 to 0.41)
**Access to PnPC team**			
No (reference)			
Do not know	0.07	0.539539	(−0.158 to 0.28)
Yes	0.30	** <0.001**	(0.14 to 0.46)
**Access to pediatric PC team**			
No (reference)			
Do not know	0.02	0.845845	(−0.21 to 0.25)
Yes	−0.15	0.086086	(−0.32 to 0.02)
**Profession**			
Physician (reference)			
Nurse	−0.31	** <0.001**	(−0.45 to −0.17)
Psychosocial staff	0.09	0.481	(−0.16 to 0.35)

*PnPC, Perinatal Palliative Care; PC, Palliative Care; P, p-value; 95%CI, 95% confidence interval; b, Unstandardized beta coefficient. The bold values indicate statistically significant p-values*.

a *Fifty-two answers excluded from multilevel multivariable analysis due to missings in variables*.

#### Perinatal Palliative Care Guidelines

Among HCPs who reported having a PnPC guideline at their center (*n* = 219), 53% reported to be satisfied, 6% dissatisfied, and 36% neither satisfied nor dissatisfied with their centers' guideline. A large majority of HCPs who reported not having or not knowing whether a PnPC guideline existed at their center (*n* = 206), expressed the need for one (80%).

Analysis of open-ended questions showed that HCPs working in a center with a guideline reported guidelines were “*too short*,” “*outdated*,” or “*not applied in practice*.” Furthermore, they wished more up-to-date and specific PnPC guidelines. HCPs further indicated that their dissatisfaction with guidelines was based on the practicality and adaptability of these guidelines to individual cases. Most HCPs reiterated that PnPC became a focal point only late into the EOL stage resulting into situations where patients would die while on the ventilator or parents were unprepared. One physician explained “*they do not focus enough on families and children, and palliative care is understood only as the termination of all measures*” (≥13 years of work experience, No. 361). Main causes for dissatisfaction with internal and external collaboration was poor communication, general unwillingness to collaborate, and lack of structural feedback mechanisms. In addition, HCPs stated that they wished guidelines would facilitate closer interdisciplinary cooperation.

## Discussion

To our knowledge, this is the first nationwide survey in Switzerland to assess PnPC from the point of view of HCPs working in tertiary NICUs. This survey found national differences in the availability of PnPC guidelines and in further PnPC training. Correspondingly, HCPs expressed the need for a PnPC guideline and further training.

### Education and Further Training

Our study shows that there remains a large group of Swiss HCPs that do not receive education in PC or further training in PnPC. As it is quite common in the Swiss healthcare system to employ HCPs from abroad (most often Germany, France and Italy) (30), we might speculate whether our findings indicate a need for better implementation of PC within the medical or nursing curriculum in other European countries as well (31–36). This explanation seems to be confirmed by the work from the European Association for Palliative Care (37, 38). In addition, the level of continued training opportunities in Swiss tertiary NICUs greatly varies across the country. Further training in PnPC is more common in the German-speaking part. These circumstances, as a result, explain variation in practice. We have shown such regional differences before in neonatal practice surrounding the end-of-life decision-making process and (39), and more specifically regarding the availability of regular staff meetings (40) and provision of consistent unit policies (41). These inconsistencies in practice might be further amplified by HCPs unawareness of the availability of further training options as it was shown in one-third of our sample.

Shortcomings in education and further training in PnPC have been widely acknowledged (3, 22, 42–45). These deficits are commonly reflected in formal aspects of medical practices and communication skills in critical settings (46–48). According to the limited data available, most PC education is offered in the form of lectures during HCPs' curriculum (49). Exposure to terminally ill patients, especially for medical or nursing students and young trainees, is limited. As a result, HCPs often feel unprepared and uncomfortable in the medical decision process or in dealing with parents (50). In recent years, education on EOL care has evolved (16, 51). Our findings show that HCPs of young age, with no children (yet), or less work experience received more PC education in their curriculum as compared to those with extensive work experience. There might have been a shift in Switzerland that includes more and earlier PC education in the curriculum. This is supported by national initiatives providing the core competencies necessary within the medical curriculum (52–54) and opportunities for HCPs to pursue continued education such as a Master of Advanced Studies or Certified Advanced Studies. In addition, gaps in the provision of palliative care have been acknowledged on a national level. In turn, the national strategy palliative care (2010–2012 and 2013–2015) aimed to improve the education for HCPs in palliative care to address financial aspects and to provide a base for increased research in this field and the public understanding of palliative care (55–57). Uniformly, it is clear that the majority of Swiss neonatal HCPs would like more training in PnPC in their centers, which would enable them to deal more confidently with palliative circumstances.

Accordingly, our findings indicate that further training within centers should incorporate PnPC in order to develop the necessary skills for the provision of high-quality PC. In line with the core competencies for palliative care of the European Association for Palliative Care (37, 38, 54), topics for further training should encompass communications skills, strengthen the ability and willingness of interdisciplinary collaboration, and promote a safe environment for HCPs and concerned parents (58). Additionally, HCPs training should be strengthened for HCPs to recognize or cope with diagnostic and prognostic uncertainty and raise the awareness for the familial background (e.g., a mother might be a patient, and proxy decision-maker or new parents might expect their first child) (38). Various studies have shown impressive success and improvement in knowledge through the introduction of simulation trainings (59, 60), multidisciplinary debriefings with external specialists, and short workshops for NICU staff (58, 61, 62). Further studies should be conducted on the implementation of PnPC educational measures that can reinforce support for PC (34, 63).

### Perinatal Palliative Care Guidelines

PnPC services and teams across Switzerland show a vast spectrum of structures and available resources. To date no national guideline on PnPC exists; instead there are some local and center-specific guidelines. However, availability of center-specific guidelines on PnPC were limited to only a few centers. Hence, our survey illustrates the issue of center-to-center variability. This has been further underlined in other studies (42, 43). Bucher et al. (21) showed national divergences in the attitudes of HCPs regarding limiting intensive care interventions for neonates. Another study showed significant impact of the medical center on survival of very low-gestational-age neonates in Switzerland (64), a disparity which may be grounded in the absence of standardized PnPC guidelines. Moreover, our study indicates a lack of national quality standards for PnPC services and teams. For example, PnPC teams currently consists of a heterogeneous mix of team structure (size, professions, knowledge, spectrum of patients and area of work) as well as resources (time, monetary, infrastructure). To date, no specialized PnPC teams with a specific defined concept and structure exist in Switzerland (65). This stands in contrast to other types of care provided in level III NICUs in which clear requirements have been defined (66). Future work needs to lay the ground works for establishing national quality standards for PnPC in Switzerland.

Variability in practice might be amplified by misunderstandings or unawareness of the availability of guidelines between interdisciplinary teams. In fact, some HCPs who had a guideline available were not always aware of this fact. On the contrary, a subset of HCPs even thought there were guidelines available when in fact there were not. Aujoulat et al. (67) similarly showed a subgroup of HCPs who were not aware of the protocols or standardized procedures regarding PnPC. This means that despite the implementation of a guideline, continued information and further training in PnPC within centers remains essential. Such continued professional education would serve the dual purpose of introducing some HCPs to the PnPC content, while advancing the awareness of others.

More importantly, HCPs who were aware of a PnPC guideline, had received further PnPC training, or had access to a PnPC team were more satisfied with PC in their center. This leads us to speculate whether the implementation of guidelines would result in less variability in practice and more staff satisfaction. In fact, evidence has repeatedly shown an improvement in the clinical practice of PnPC with standardized guidelines (68–71). This supports the view that PnPC services can facilitate palliative care (34, 63). In addition, our findings show that a large majority of HCPs, who disclosed not having or not knowing about a guideline at their center, expressed the need for one. Hence, national recommendations are necessary to minimize center-to-center variability, and thereby to limit conflicting and ambiguous practice of care. This might eventually result in less moral distress for HCPs and potentially lead to more confidence and satisfaction in dealing with these emotionally loaded situations in PnPC.

Interestingly, nurses were more likely to be dissatisfied with the PnPC guidelines than physicians or psychosocial staff. Several interpretations can be made. In general, nurses spend more time at the patients' bedside and with families. This can impact their perceptions as they might be more often confronted both with parental demands and dissatisfaction (39, 72), and with institutional barriers to providing high quality PnPC. The open-ended answers also suggest a difference in the nurses' and physicians' perspective with physicians frequently equating PC to EOL. Evidence shows that such misinterpretation denies patients the opportunity to appropriate treatment (34). Knowing that nurses in our study had more PC education, physicians' misinterpretation of PnPC might have led to dissatisfaction among nursing staff. This further shows the importance of establishing multidisciplinary PnPC teams that include all perspectives from a range of disciplines involved.

According to HCPs working in Swiss tertiary NICUs, local PnPC guidelines mainly focus on parental needs in the form of psychosocial support and pastoral care. With the exception of comfort related concerns, pain management and treatment of other neonatal symptoms of distress are, surprisingly, less often mentioned. Nevertheless, pain management plays an important role in PnPC that requires repeated re-evaluation and adaptation (73). Future studies should further assess the content of center-specific guidelines on completeness of essential topics necessary to a high-quality PnPC. It might even be possible that in our surveyed sample PnPC guidelines were not well-known among some HCPs because its content was incomplete or outdated. Such factors could potentially lead to further dissatisfaction in care. Hence, not only does every NICU need a guideline; it is also necessary to formulate quality standards, which can be achieved by developing national recommendations. The introduction of a national guideline, through interdisciplinary exchange involving physicians, nurses and psychosocial staff members, might facilitate the implementation of PnPC first on a national and then on local levels. Subsequently, such a national guideline may well serve as a framework for local endeavors. This allows local processes to emerge while respecting a national consensus.

### Limitations

A number of limitations need to be considered. The response rate of 54%, although not atypical (16, 74, 75), raises a concern regarding generalizability of the results. This response rate may be due to a high number of survey requests and/or lack of voucher incentives (76, 77). However, the distribution of participants' professions taking part in our survey represents the proportions found in Swiss tertiary NICUs as shown in other studies by the SN-EOL Study Group. This allows us to extrapolate the collected information to the larger population of Swiss neonatal HCPs (21, 40). Future assessments of PnPC should include the viewpoints of perinatal HCPs such as feto-maternal physicians, obstetricians and midwives, which were not sampled in this study. Furthermore, our results are based on the self-reports of participants, not on direct observations. We have aimed to overcome this limitation by requesting the centers for their internal PnPC guidelines. Furthermore, we only investigated the existence and knowledge of PnPC guidelines; we did not, however, examine whether center-specific guidelines were (in)complete regarding essential PnPC topics. Finally, the purpose of the third aim of our study was to provide insights in what can be improved, hence the negative impressions of HCPs might be amplified by looking through the lens of what needs improving, rather than explaining what works well. Notwithstanding the survey's limitations, our nationwide survey adds to the hitherto limited knowledge of PnPC practices.

## Conclusion

Our results reveal a lack of consistent PnPC guidelines and continued training across Swiss level III NICUs. HCPs' expressed the need for guidelines to be formulated and demanded more opportunities for continuing professional education and training in their working environment. Neonatal HCPs who were aware of guidelines, who received further PnPC training, and who had access to PnPC teams reported greater satisfaction with the provided PC. Extending such PnPC services to all centers can help bridge the barriers created by fragmented practice. A standardized national guideline may help in providing high-quality PnPC services consistently to critically ill patients and families and might help facilitate local implementation of PnPC.

## Data Availability Statement

The datasets presented in this article are available from the corresponding author [MH], upon reasonable request.

## Ethics Statement

The studies involving human participants were reviewed and approved by The Ethics Commission of the Canton of Zurich (Study ID: 2019-00176). The patients/participants provided their written informed consent to participate in this study.

## Author Contributions

The study was conceived by MH, DG, and J-CF. MH and DG acquired funding. Data was collected by AB and supervised by MH and DG. Data was analyzed by AB, MH, and JB and interpreted by all authors. AB made the first draft. All authors contributed to the critical revision, approved the final manuscript, have sufficiently participated in the work to take public responsibility for appropriate portions of the content, and approve the version for publication.

## Conflict of Interest

The authors declare that the research was conducted in the absence of any commercial or financial relationships that could be construed as a potential conflict of interest.

## Supplementary Material

Supplementary Data Sheet 1 Supplementary Table 1.

Supplementary Data Sheet 2 Supplementary Figure 1.

Supplementary Data Sheet 3 Questionnaire.

Supplementary Data Sheet 4 Appendix.

## Abbreviations

CI: Confidence interval
EOL: End-of-life
HCPs: Healthcare professionals
NICU: Neonatal intensive care unit
PnPC: Perinatal Palliative Care
PC: Palliative care.
