# Supplementary material for: National Divergences in Perinatal Palliative Care Guidelines and Training in Tertiary NICUs
Source: Front Pediatr. 2021 Jul 14;9:673545. doi: 10.3389/fped.2021.673545 (PMC8316587; doi:10.3389/fped.2021.673545)
Supplement: Supplementary Data Sheet 1 — Supplementary Table 1. [file Data_Sheet_1.docx]

**Supplementary Table 1**. Associations between healthcare professionals’ characteristics and received PC education or PnPC training

|  | **Total Participants**  **N= 436** | **Participants with PC education in curriculum**  **N=217** | | |  | **Participants with PnPC further training at center**  **N=158** | | |
| --- | --- | --- | --- | --- | --- | --- | --- | --- |
|  | **n (%)** | **n** | **%** | **p-Value** |  | **n** | **%** | **p-Value** |
|  |  |  |  |  |  |  |  |  |
| **Language region** |  |  |  |  |  |  |  |  |
| German | 365 (83.7) | 174 | 47.7 | 0.135 |  | 148 | 40.5 | **<0.001** |
| French | 71 (16.3) | 43 | 60.6 |  |  | 10 | 14.1 |  |
| **Gender** |  |  |  |  |  |  |  |  |
| Male | 52 (11.9) | 21 | 41.2 | 0.680 |  | 22 | 42.3 | 0.418 |
| Female | 382 (87.6) | 195 | 52.3 |  |  | 135 | 35.3 |  |
| Other | 2 (0.5) | 1 | 50.0 |  |  | 1 | 50.0 |  |
| **Age at study** |  |  |  |  |  |  |  |  |
| < 30 years | 75 (17.2) | 52 | 69.3 | **<0.001** |  | 23 | 30.7 | **0.001** |
| 30-39 years | 151 (34.6) | 94 | 62.3 |  |  | 49 | 32.5 |  |
| 40-49 years | 119 (27.3) | 53 | 44.5 |  |  | 45 | 37.8 |  |
| > 50 years | 91 (20.9) | 18 | 19.8 |  |  | 41 | 45.1 |  |
| **Own children^a,b^** |  |  |  |  |  |  |  |  |
| Yes | 216 (49.5) | 91 | 42.1 | **0.007** |  | 88 | 40.7 | 0.168 |
| No | 218 (50.0) | 126 | 57.8 |  |  | 70 | 32.1 |  |
| Not filled in (i.e. missing) | 2 (0.5) | 0 |  |  |  | 0 |  |  |
| **Religion** |  |  |  |  |  |  |  |  |
| Catholic | 153 (35.1) | 78 | 50.1 | 0.144 |  | 57 | 37.3 | 0.162 |
| None | 148 (33.9) | 74 | 50.0 |  |  | 49 | 33.1 |  |
| Protestant | 114 (26.2) | 52 | 45.6 |  |  | 49 | 42.9 |  |
| Muslim | 6 (1.4) | 4 | 66.7 |  |  | 0 | 0 |  |
| Other | 15 (3.4) | 9 | 60.0 |  |  | 3 | 20.0 |  |
| **Profession** |  |  |  |  |  |  |  |  |
| Physician | 106 (24.3) | 40 | 37.7 | **<0.001** |  | 30 | 28.3 | 0.164 |
| Nurse | 297 (68.1) | 171 | 57.6 |  |  | 111 | 37.4 |  |
| Psychosocial staff | 33 (7.6) | 6 | 18.2 |  |  | 17 | 51.5 |  |
| **Leading position (n=403)^a,b^** |  |  |  |  |  |  |  |  |
| Yes | 103 (29.2) | 34 | 33.0 | **<0.001** |  | 42 | 40.8 | 0.166 |
| No | 250 (70.8) | 146 | 58.4 |  |  | 78 | 31.2 |  |
| Not filled in (i.e. missing) | 50 (12.4) | 37 |  |  |  | 38 |  |  |
| **Country of training** |  |  |  |  |  |  |  |  |
| Switzerland | 326 (74.7) | 160 | 49.1 | 0.805 |  | 121 | 37.1 | 0.432 |
| Abroad | 110 (25.3) | 57 | 51.8 |  |  | 37 | 33.6 |  |
| **Working experience** |  |  |  |  |  |  |  |  |
| Little work experience (≤ 6 years) | 150 (34.4) | 94 | 62.7 | **<0.001** |  | 43 | 28.7 | **0.002** |
| Moderate work experience (7-12 years) | 112 (25.7) | 62 | 55.4 |  |  | 43 | 38.4 |  |
| Extensive work experience (≥ 13 years) | 174 (39.9) | 61 | 35.1 |  |  | 71 | 41.4 |  |
| ^a^ chi-squared testing does not include those with missing  ^b^ Physicians and nurses only  Abbreviations: PC, Palliative Care; PnPC, Perinatal palliative care | | | | | | | | |
