# Supplementary material for: National Divergences in Perinatal Palliative Care Guidelines and Training in Tertiary NICUs
Source: Front Pediatr. 2021 Jul 14;9:673545. doi: 10.3389/fped.2021.673545 (PMC8316587; doi:10.3389/fped.2021.673545)
Supplement: Supplementary Data Sheet 2 — Supplementary Figure 1. [file Data_Sheet_2.docx]

Supplementary Material


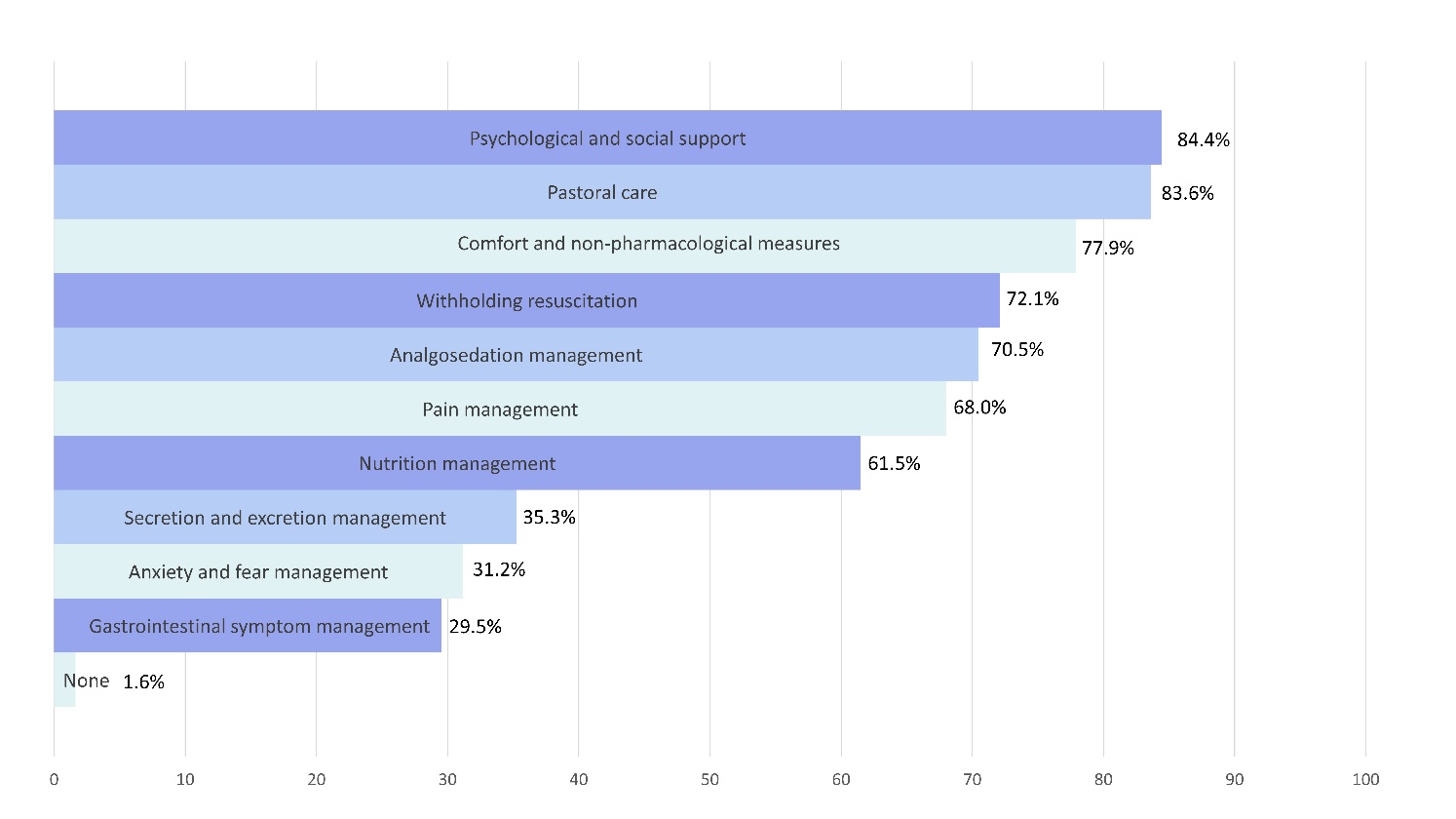


**Supplementary Figure 1.** Content of Swiss perinatal palliative care guidelines
