# Supplementary material for: National Divergences in Perinatal Palliative Care Guidelines and Training in Tertiary NICUs
Source: Front Pediatr. 2021 Jul 14;9:673545. doi: 10.3389/fped.2021.673545 (PMC8316587; doi:10.3389/fped.2021.673545)
Supplement: Supplementary Data Sheet 3 — Questionnaire. [file Data_Sheet_3.DOCX]

**Supplementary Material 1**. The healthcare professionals on perinatal palliative care (HCP-PerinatPC) Survey in Swiss neonatal intensive care units^[[1]](#footnote-1)^

**EL01**

**F.0**

Good morning

We invite you to participate in the following survey: "Perinatal Palliative Care in Switzerland". The importance and urgency of this topic is partly due to the results of the NRP67 research 'End of life', in which you may have participated. This survey is aimed at health care professionals at the neonatology department in perinatal centers throughout Switzerland.

**EL02
F.1a Do you agree to fill out the following questionnaire?**

It will take about 10-15 minutes to complete.

1 🔿 yes

2 🔿 no (logical branch/filter to: EL04)

**EL04
F.2**

**Please tell us your reasons:***(multiple answers possible)*

1 🔿 because I have no time

2 🔿 because I am not interested in the topic

3 🔿 other: ............................................................

4 🔿 no reasons

### Part A

| **SD01 First of all, we would like to know something about your professional background.** | |
| --- | --- |
| **SD02 F.3** **How long have you been working in obstetrics or neonatology?**  *(Please also count the time you have worked in your department in another hospital. In case of interruptions, please add up the respective periods of time and indicate the total time)*  1 🔿 less than 1 year  2 🔿 1-6 years  3 🔿 7-12 years  4 🔿 13-20 years  5 🔿 more than 20 years | |
| **SD05 F.4 What is your profession?**  1 🔿 physician  2 🔿 nurse  3 🔿 others: (z.B. psychologist, psychiatrist, pastor)____________________  (logical branch/filter to: VS03-04, VS07, VS09, VS12-14, VS22-23, VS30) | **SD06**  **F.5 In which area do you work?**  1 🔿 Obstetrics  2 🔿 Neonatology (logical branch/filter to: SD09, VS13-14) |
| **SD15**  **F.6 In which unit do you work?**  1 🔿 Delivery room  2 🔿 Postpartum bed  3 🔿 Neonatal Intensive Care Unit (NICU)  4 🔿 Neonatology ward | |

| **SD07**  **F.7 Do you have a leading position?**  (e.g. chief physician; head physician; senior physician; head of nursing; head of nursing ward; head of nursing group; head of nursing group; nursing expert in a leading position; head of the midwife team)  1 🔿 yes  2 🔿 no |
| --- |
| **SD08**  **F.8 In which country (countries) were you trained?**  *(multiple answers possible)*  1 ❒ Switzerland  2 ❒ Germany  3 ❒ France  4 ❒ Italy  5 ❒ Austria  6 ❒ others (please name): ....................................................... |
| **SD09**  **F.9 Are you involved in the follow-up care of newborns after discharge from neonatology? If so, how long?** *(Please add to the number whether it concerns days, weeks or months)*  1 🔿 yes :_____________  2 🔿 no |
| **SD10 F.10 Your Gender:**  1 🔿 male  2 🔿 female  3 🔿 _________ |

| **SD11**  **F.11 Your age:**  1 🔿 younger than 20  2 🔿 20-29  3 🔿 30-39  4 🔿 40-49  5 🔿 50 or above |
| --- |
| **SD12**  **F.12 Do you have children?**  1 🔿 yes  2 🔿 no |
| **SD13**  **F.13 What is your religion?**  1 🔿 none  2 🔿 catholic  3 🔿 reformed  4 🔿 jewish  5 🔿 muslim  6 🔿 others: ____________________ |
| **SD14 F.14 How important is your spirituality/religion to you in your everyday life?**  1 🔿 very important  2 🔿 rather important  3 🔿 partly important  4 🔿 barely important  5 🔿 not important |

### Part B

| **We would like to hear your views on palliative care of newborn children. Please only express your personal opinion and not the opinions and procedures represented on your ward.** |
| --- |
| **AP02 F.15 How important is your spirituality/religion to you in relation to the palliative care of newborns in your department?**  1 🔿 very important  2 🔿 rather important  3 🔿 partly important  4 🔿 barely important  5 🔿 not important |
| **AP03 F.16 How much does your own view/attitude influence the counseling of families with regard to decision-making in critical situations that also affect the end of life?**  1 🔿 very strong  2 🔿 strong  3 🔿 medium  4 🔿 weak  5 🔿 not at all |

### Part C

| **We would like to know more about the specific care process in your department regarding palliative care of newborn children.** |
| --- |
| **VS03 F.17 How many preterm infants with extremely low birth weight (<1000g)/extremely low gestational age (22-27 SSW) do you think are admitted to your department each month?** *Estimation question (no precise information necessary)*  Number: ____________________________  99 🔿 do not know |
| **VS04**  **F.18 How many newborns with complex malformations (affecting more than one organ system) do you think are admitted to your department each month?** *Estimation question (no precise information necessary)*  Number: ____________________________  99 🔿 do not know |
| **VS05**  **F.19 How many newborns with a palliative diagnosis have you treated in your department in the last year?**  99 🔿 none  1 🔿 less than 5  2 🔿 around 5-10  3 🔿 around 10-15  4 🔿 more than 15 |
| **VS06**  **F.20 Was palliative care offered as a topic in your studies / school curriculum?**  1 🔿 yes  2 🔿 no |

| **VS07**  **F. 21** **Does your institution offer further education and training in perinatal palliative care?**  1 🔿 yes (logical branch/filter to: VS08)  2 🔿 no  99 🔿 do not know |
| --- |
| **VS08**  **F. 22 If your institution offers continuing education in perinatal palliative care, what does it include?** *(multiple responses possible)*  1 🔿 in-house training courses  2 🔿 web seminars  3 🔿 lectures  4 🔿 workshops  6 🔿 others: ____________________ |
| **VS09 F.23 Does your institution have a standardized prescription set for perinatal palliative care in the electronic patient file?**  1 🔿 yes (logical branch/filter to: VS11)  2 🔿 no  99 🔿 Do not know |
| **VS10**  **F.24 Does your institution have guidelines for perinatal palliative care?**  1 🔿 yes (logical branch/filter to: VS11, ZP04, ZP06)  2 🔿 no (logical branch/filter to: VS12, ZP07, ZP12)  99 🔿 do not know (logical branch/filter to: VS12, ZP07, ZP12) |

| **VS11**  **F.25** **If there is a standardized prescription set and/or guidelines for perinatal palliative care of newborns in your department, what do they include?**  *Please click all applicable items (multiple answers possible)*  1 🔿 disease-related pain or discomfort  2 🔿 nutrition of newborns  3 🔿 secretion/excretion  4 🔿 symptom management of gastrointestinal problems  5 🔿 non-pharmacological measures to reduce pain/increase comfort (primarily physical contact, ideally with parents)  6 🔿 symptom management of anxieties Angst  7 🔿 pharmacological analgosedation (Fentanyl intranasal, other opiates)  8 🔿 renunciation of resuscitation  9 🔿 psychological/social support for relatives  10 🔿 pastoral care  11 🔿 none of the above  99 🔿 do not know |
| --- |
| **VS30**  **F.26 Are analgosedating drugs administered?**  1 🔿 yes (logical branch/filter to: VS22-23)  2 🔿 no  99 🔿 do not know |
| **VS22**  **F.27 How often is pharmacological analgosedation administered in palliative situations for symptom control?**  1 🔿 always  2 🔿 often  3 🔿 sometimes  4 🔿 rarely  5 🔿 never |
| **VS23**  **F.28 Analgosedative drugs for symptom control are administered because of:**  *(multiple answers possible)*  1 🔿 indications given by neonate  2 🔿 indications given by parents |
| **VS12**  **F.29 If the policy of your institution deals with nutrition, which of the following are offered?**  1 🔿 breast feeding  2 🔿 nasogastric or orogastric tube feeding  3 🔿 intravenous fluid supply  4 🔿 other: ____________________  5 🔿 none of the above  99 🔿 do not know |
| **VS13 F.30 Which of the following medical life support measures are offered to palliative patients at their workplace?**  1 🔿 gastric tube for fluid supply  2 🔿 gastric tube for nutrition  3 🔿 gastrostomy probe for fluid supply  4 🔿 gastrostomy probe for nutrition  5 🔿 intravenous fluid supply  6 🔿 catecholamine  7 🔿 invasive or non-invasive ventilation  8 🔿 dialysis or continuous renal replacement therapy  9 🔿 transplantation  10 🔿 ECMO (extracorporal membrane oxygenastion)  11 🔿 none of the above  99 🔿 do not know |

| **VS14**  **F.31 Is it reasonable in your institution to withhold nutrition (i.v., by stomach tube, gastrostomy) from a patient with a palliative diagnosis?**  1 🔿 yes  2 🔿 no  99 🔿 do not know |
| --- |
| **VS15 F.32 Do you have access to a perinatal/neonatal palliative care team in your institution?**  1 🔿 yes  2 🔿 no  99 🔿 do not know |
| **VS16**  **F.33 Do you have access to a pediatric palliative care team in your institution?**  1 🔿 yes  2 🔿 no  99 🔿 do not know |
| **VS24**  **F.34 Who is involved in the palliative care of a newborn in your hospital?**  *(multiple answers possible)*  1 🔿 reference nurse  2 🔿 family doctor  3 🔿 midwives  4 🔿 obstetrician  5 🔿 pediatrician  6 🔿 neonatologist  7 🔿 psychologist  8 🔿 psychiatrist  9 🔿 pastor  10 🔿 others: _____________  99 🔿 do not know |
| **VS25**  **F.35 Are parents also offered the possibility of palliative care at home?**  1 🔿 yes (logical branch/filter to: VS26)  2 🔿 no  99 🔿 do not know |
| **VS26**  **F.36 Who is involved in the palliative care of a newborn from home?**  *(multiple options possible*  1 🔿 reference nurse  2 🔿 family doctor  3 🔿 midwives  4 🔿 obstetrician  5 🔿 pediatrician  6 🔿 neonatologist  7 🔿 psychologist  8 🔿 psychiatrist  9 🔿 pastor  10 🔿 home care  11 🔿 others: _____________  99 🔿 do not know |
| **VS17**  **F.37 Does your institution work with other organizations that care for newborns with a palliative diagnosis?** *Please name them (e.g. home care, volunteer organizations, children’s home,…)*  1 🔿 yes:___________________  2 🔿 no  99 🔿 do not know |

| **VS18**  **F.38 Does your institution have an ethics committee?**  1 🔿 yes  2 🔿 no  99 🔿 do not know |
| --- |
| **VS19**  **F.39 Does your institution provide families with brochures/literature on palliative care for newborns?**  1 🔿 yes  2 🔿 no  99 🔿 do not know |
| **VS20**  **F.40 Does your institution offer grief counselling for families?**  1 🔿 yes (logical branch/filter to: VS21)  2 🔿 no  99 🔿 do not know |
| **VS21**  **F.41 If grief counseling is offered, who is primarily responsible?**  *(multiple options possible)*  1 🔿 reference nurse  2 🔿 family doctor  3 🔿 midwives  4 🔿 obstetrician  5 🔿 pediatrician  6 🔿 neonatologist  7 🔿 psychologist  8 🔿 psychiatrist  9 🔿 pastor  10 🔿 nursing responsible for grief counseling  11 🔿 others: ____________________  99 🔿 do not know |
| **VS27 F.42 Does your institution offer a follow-up meeting for families?**  1 🔿 Yes (logical branch/filter to: VS28-29)  2 🔿 No  99 🔿 do not know |
| **VS28 F.43 If a follow up is offered, who is responsible?**  *(multiple options possible)*  1 🔿 reference nurse  2 🔿 family doctor  3 🔿 midwives  4 🔿 obstetrician  5 🔿 pediatrician  6 🔿 neonatologist  7 🔿 psychologist  8 🔿 psychiatrist  9 🔿 pastor  10 🔿 others: ____________________  99 🔿 do not know |
| **VS29 F.44 If a follow-up call is offered, are the calls protocolled?**  1 🔿 yes  2 🔿 sometimes  3 🔿 no  99 🔿 do not know |

### Part D

| **We would like to question you about your satisfaction with palliative care on your ward.** |
| --- |
| **ZP11 F.45 Are you satisfied with the palliative care on your ward?**  *(Please explain your stance)*  1 🔿 yes, because:__________________________________  2 🔿 partly, because:____________________________  3 🔿 no, because:________________________________ |
| **ZP02**  **F.46 Does your institution have an offer for coping with mental stress for the care team?**  1 🔿 yes (logical branch/filter to: ZP03)  2 🔿 no  99 🔿 do not know |
| **ZP03 F.47 If yes, do you take up this offer?**  1 🔿 yes  2 🔿 no |
| **ZP04**  **F.48**  **Overall, how satisfied are you with your institution's guidelines for perinatal palliative care?**  1 🔿 very satisfied  2 🔿 satisfied  3 🔿 partly satisfied/partly dissatisfied  4 🔿 dissatisfied (logical branch/filter to: ZP12)  5 🔿 very dissatisfied (logical branch/filter to: ZP12) |
| **ZP12**  **F.49 Why are you dissatisfied with the guidelines of your institution?**  ________________________________________________________ |
| **ZP05 F.50 Do you think that your institution would benefit from more education/training in perinatal palliative care?**  1 🔿 yes  2 🔿 no |
| **ZP09**  **F.51 How satisfied are you with the interprofessional collaboration in the hospital in regards to perinatal palliative care ?**  1 🔿 very satisfied  2 🔿 satisfied  3 🔿 partly satisfied/partly dissatisfied  4 🔿 dissatisfied (logical branch/filter to: ZP13)  5 🔿 very dissatisfied (logical branch/filter to: ZP13) |
| **ZP10 F.52 How satisfied are you with the interprofessional cooperation with external specialists for perinatal palliative care?**  1 🔿 very satisfied  2 🔿 satisfied  3 🔿 partly satisfied/partly dissatisfied  4 🔿 dissatisfied (logical branch/filter to: ZP13)  5 🔿 very dissatisfied (logical branch/filter to: ZP13) |
| **ZP13**  **F.53 Why are you dissatisfied with interprofessional cooperation?**  ________________________________________________________ |
| **ZP07 F.54 If there are no guidelines in your institution, would you like one?**  1 🔿 yes  2 🔿 no |

| **ZP08 F.55 Are there any other aspects in this context that you would like to share with us?**                      ❒ No, I do not want to add/supplement anything more. |
| --- |

**Thank you very much for your participation in this survey!**

1. The HCP-PerinatPC Survey is available in French and German. Contact the corresponding author for more information. [↑](#footnote-ref-1)
