# Supplementary material for: National Divergences in Perinatal Palliative Care Guidelines and Training in Tertiary NICUs
Source: Front Pediatr. 2021 Jul 14;9:673545. doi: 10.3389/fped.2021.673545 (PMC8316587; doi:10.3389/fped.2021.673545)
Supplement: Supplementary Data Sheet 4 — Appendix. [file Data_Sheet_4.DOCX]

Supplementary Material

# SUPPLEMENTARY MATERIAL 2. Appendix

## Members of the Swiss Neonatal End-of-Life Study Group (listed in alphabetical order of study site):

Aarau: Neonatal Unit, Dept. of Pediatrics, Kantonsspital Aarau (Meyer Philipp, MD; Celine Gautier) – Basel: Neonatal Unit, University Children’s Hospital Basel UKBB (Neumann Roland, MD; Itin Renate) – Bern: Neonatal Unit, University Children’s Hospital, Inselspital (Humpl Tilman, MD; Stoffel Liliane) – Chur: Neonatal Unit, Dept. of Paediatrics, Kantonsspital Chur (Scharrer Brigitte, MD; Roloff Kai) – Geneva: Neonatology and Pediatric Intensive Care, Dept. of Paediatrics, University Hospital HCUG (Pfister Riccardo, MD) – Lausanne: Division of Neonatology, Dept. of Paediatrics, University Hospital CHUV (Truttmann Anita, MD; Contino Magali) – Lucerne: Neonatal Unit, Children’s Hospital, Kantonsspital Luzern (Schwendener Katharina, MD; Stalder Sandra) – St. Gallen: Neonatal Unit, Children’s Hospital, Kantonsspital St. Gallen (Jaeger Gudrun, MD; Dutler Ruth) – Zurich: Department of Neonatology, University Hospital Zurich (Fauchère Jean-Claude, MD; Dinten Barbara)
